# Fusion transcript loci share many genomic features with non-fusion loci

**DOI:** 10.1186/s12864-015-2235-4

**Published:** 2015-12-01

**Authors:** John Lai, Jiyuan An, Inge Seim, Carina Walpole, Andrea Hoffman, Leire Moya, Srilakshmi Srinivasan, Joanna L. Perry-Keene, Australian Prostate Cancer Bioresource, Chenwei Wang, Melanie L. Lehman, Colleen C. Nelson, Judith A. Clements, Jyotsna Batra

**Affiliations:** Australian Prostate Cancer Research Centre – Queensland, Translational Research Institute, Brisbane, Australia; Cancer and Molecular Medicine Program, Institute of Health and Biomedical Innovation, Queensland University of Technology, Brisbane, Australia; Comparative and Endocrine Biology Laboratory, Institute of Health and Biomedical Innovation, Brisbane, Australia; Ghrelin Research Group, Institute of Health and Biomedical Innovation, Brisbane, Australia; Anatomical Pathology, Pathology Queensland, Brisbane, Australia; Current address: Genetic Technologies, 60-66 Hanover Street, Melbourne, Australia

**Keywords:** Fusion, RNA, RNAseq, Prostate cancer

## Abstract

**Background:**

Fusion transcripts are found in many tissues and have the potential to create novel functional products. Here, we investigate the genomic sequences around fusion junctions to better understand the transcriptional mechanisms mediating fusion transcription/splicing. We analyzed data from prostate (cancer) cells as previous studies have shown extensively that these cells readily undergo fusion transcription.

**Results:**

We used the FusionMap program to identify high-confidence fusion transcripts from RNAseq data. The RNAseq datasets were from our (*N* = 8) and other (*N* = 14) clinical prostate tumors with adjacent non-cancer cells, and from the LNCaP prostate cancer cell line that were mock-, androgen- (DHT), and anti-androgen- (bicalutamide, enzalutamide) treated. In total, 185 fusion transcripts were identified from all RNAseq datasets. The majority (76 %) of these fusion transcripts were ‘read-through chimeras’ derived from adjacent genes in the genome. Characterization of sequences at fusion loci were carried out using a combination of the FusionMap program, custom Perl scripts, and the RNAfold program. Our computational analysis indicated that most fusion junctions (76 %) use the consensus GT-AG intron donor-acceptor splice site, and most fusion transcripts (85 %) maintained the open reading frame. We assessed whether parental genes of fusion transcripts have the potential to form complementary base pairing between parental genes which might bring them into physical proximity. Our computational analysis of sequences flanking fusion junctions at parental loci indicate that these loci have a similar propensity as non-fusion loci to hybridize. The abundance of repetitive sequences at fusion and non-fusion loci was also investigated given that SINE repeats are involved in aberrant gene transcription. We found few instances of repetitive sequences at both fusion and non-fusion junctions. Finally, RT-qPCR was performed on RNA from both clinical prostate tumors and adjacent non-cancer cells (*N* = 7), and LNCaP cells treated as above to validate the expression of seven fusion transcripts and their respective parental genes. We reveal that fusion transcript expression is similar to the expression of parental genes.

**Conclusions:**

Fusion transcripts maintain the open reading frame, and likely use the same transcriptional machinery as non-fusion transcripts as they share many genomic features at splice/fusion junctions.

**Electronic supplementary material:**

The online version of this article (doi:10.1186/s12864-015-2235-4) contains supplementary material, which is available to authorized users.

## Background

The latest estimates indicate that the human genome comprises only 20,687 protein coding genes [1]. This number seems surprisingly low, considering the phenotypic complexity of humans. Adding another layer of complexity, it is now appreciated that fusion transcripts -- which are derived of exons from two or more distinct genes -- can exponentially increase the protein coding/functional capacity of the human genome [2]. There is now a body of evidence to indicate that numerous genes within the human genome are transcribed as fusion transcripts [3–5]. Notably, some fusion transcripts are more tissue specific than non-fusion transcripts, and are translated into proteins [5].

Here, we use prostate (cancer) cells as a model to study fusion transcription given extensive studies that indicate that the prostate readily expresses fusion transcripts. For example, the most studied fusion in prostate cancer is formed between the *TMPRSS2* and *ERG* genes, resulting in *ERG* transcription being driven by the androgen-responsive *TMPRSS2* promoter [6–8]. This fusion is observed in ~50 % of primary prostate tumors, and ~41 % of lymph node metastatic tumors [8]. Hundreds of novel fusion genes that are formed by chromosomal rearrangements have since been discovered in prostate cancer genomes [9, 10]. Interestingly, some of these chromosomal rearrangement fusion genes can produce fusion transcripts comprising exons from more than two genes [11]. The Chinnaiyan laboratory extended their seminal *TMPRSS2-ERG* study by using RNAseq to identify 11 other fusion transcripts that are not produced by chromosomal alterations (hereafter termed transcription-induced transcripts) [12, 13]. Later studies using RNAseq estimated that there may be as many as 339 transcription-mediated fusion transcripts that are expressed in the prostate [14]. Importantly, Maher and colleagues revealed that some transcription-mediated fusion transcripts such as *SLC45A3-ELK4* are more highly expressed in metastatic prostate cancers compared to benign cells [12]. Other studies [15, 16] have since correlated *SLC45A3-ELK4* expression with an unfavorable prostate cancer prognosis, resulting in a growing interest in fusion transcription in the prostate cancer biomarker field [17, 18].

A recent study of 974 diverse cancer cases has identified 198 fusion transcripts, some of which comprise kinase genes that have great potential to be targeted therapeutically [9]. Additionally, a more recent extensive study of 7256 RNAseq libraries discovered 8020 transcription-mediated fusion transcripts, many of which are expressed in the prostate and/or associated with various types of cancer [19]. Interestingly, fusion transcripts have also been found to be formed between mitochondrial DNA with nuclear DNA, occurring at a similar frequency as fusion transcripts that comprise solely of nuclear DNA [20].

In this study we characterized the genomic sequences flanking fusion transcripts to better understand the mechanisms that mediate fusion transcription, using prostate (cancer) as a model given the aforementioned extensive studies in this tissue. Indeed, a study in prostate (cancer) cells reveals that the CTCF transcription factor mediates changes in chromosomal conformation that results in the possible formation of at least 56 fusion transcripts {Qin, 2015 #33}. Here, we reveal that the sequences flanking fusion loci are similar to non-fusion loci, indicating that the mechanisms adopted by fusion transcription are likely to be similar to non-fusion transcription and intron splicing.

## Results and discussion

### Identification of fusion transcripts in prostate cancer

A recent study indicates that the number of protein coding genes in the human genome is similar to lower vertebrates [21]. Thus, there has been a growing interest in fusion transcription as a mechanism to account for some of the phenotypic complexities of humans [2]. Here, we used the FusionMap program to first identify fusion transcripts in prostate (cancer) RNA-seq data sets as this program offers one of the best compromises between sensitivity and false positives [22]. Predicted fusion transcripts were then searched against the genome using the BLAT function of the UCSC genome browser, and manually inspected to filter out predicted fusion transcripts that map to other regions of the genome (false positives).

**Fig. 1:**
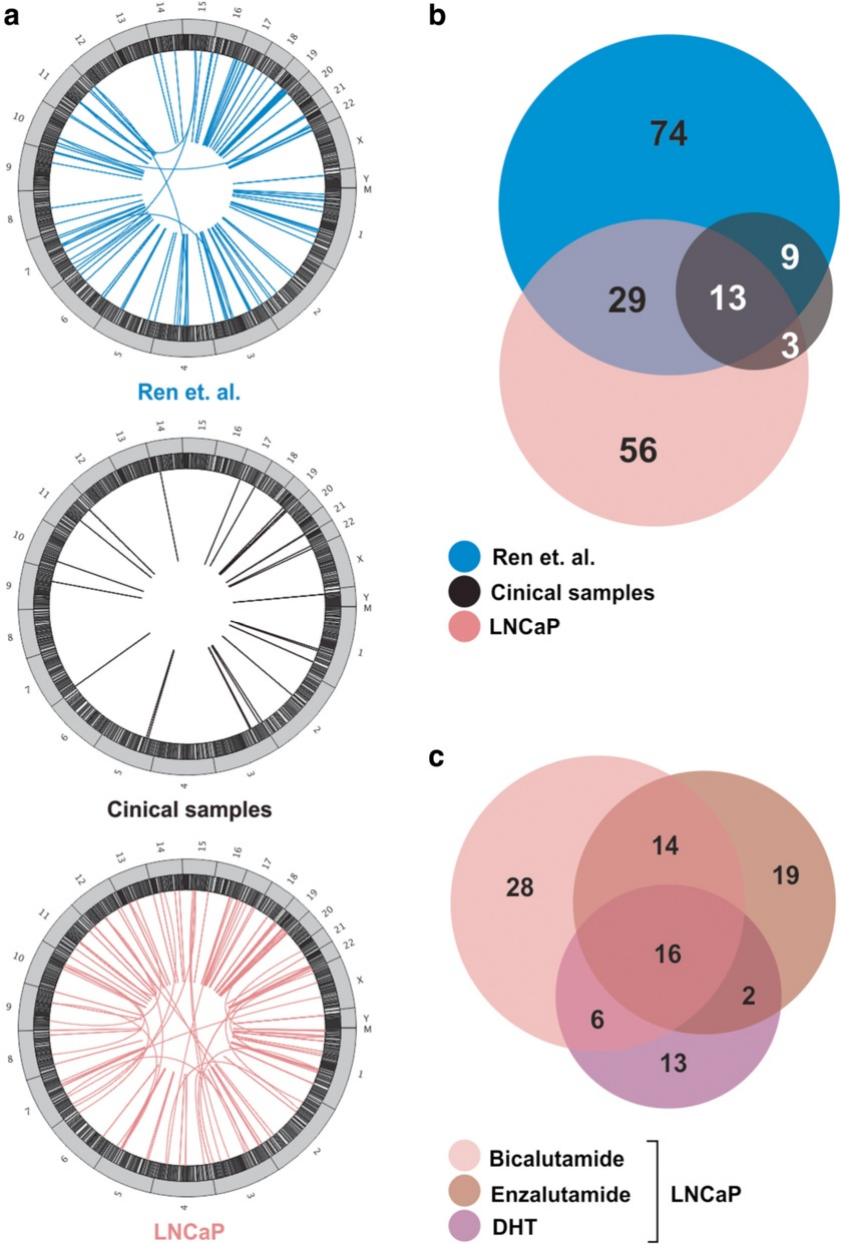
**a** Circos plot from RNAseq data of fusion transcripts from the Ren et. al. dataset [29], from our clinical prostate cancers and adjacent non-cancer prostate cells (*n* = 8), and from LNCaP prostate cancer cells that were treated with either 10 nM androgen (DHT) or 10 μM anti-androgen (bicalutamide and enzalutamide). **b** Venn diagram detailing how many unique fusion transcripts were detected between the different RNAseq datasets. **c** Venn diagram detailing how many unique fusion transcripts were detected between androgen or anti-androgen treated LNCaP cells

This resulted in the detection of a total of 185 high-confidence fusion transcripts from Ren and colleagues (14 patients) and our (eight patients) dataset of clinical prostate cancers and their adjacent non-cancer prostate cells, and from our dataset of cultured LNCaP cells treated with androgens (DHT) and anti-androgens (bicalutamide and enzalutamide) (Additional file 1). The majority of these fusion transcripts (140/185, 76 %) are derived from genes that are located next to each other in the genome, otherwise referred to as “read-through transcripts” [13], or transcription induced chimeras [23, 24] (Additional file 1). This observation is supported by a recent study in prostate cancer cells that indicates that a high percentage of fusion transcripts involve neighbouring genes {Qin, 2015 #33}. Of the other fusion transcripts, 15 (8 %) are derived from genes that are located on different chromosomes, and 30 (16 %) are derived from non-adjacent genes that are on the same chromosome (Fig. 1a and Additional file 1). Notably, a majority of fusion transcripts were solely detected in either Ren and colleagues (74 fusions, 40 %) or the LNCaP (56 fusions, 30 %) datasets (Fig. 1b), and some fusion transcripts were exclusively detected in LNCaP cells that were treated with either bicalutamide (28 fusions, 29 %), enzalutamide (19 fusions, 19 %), or DHT (13 fusions, 13 %) (Fig. 1c). This highlights the importance of using many different biological data sets to identify fusion transcripts.

### Most fusion transcripts are formed at annotated exon junctions, use classical GT-AG intron donor-acceptor motifs, and preserve the open reading frame

Previous studies have characterized fusion transcript splice sites at the exon usage level [3] or at the RNA sequence level [4], but to our knowledge, there has been no attempt to characterize the genomic sequence surrounding fusion junctions. Thus, we have characterized the nucleotide sequences flanking fusion junctions at the genes that fusion transcripts are derived (hereafter referred to as parental genes) to better understand the mechanisms that mediate fusion expression.

**Fig. 2:**
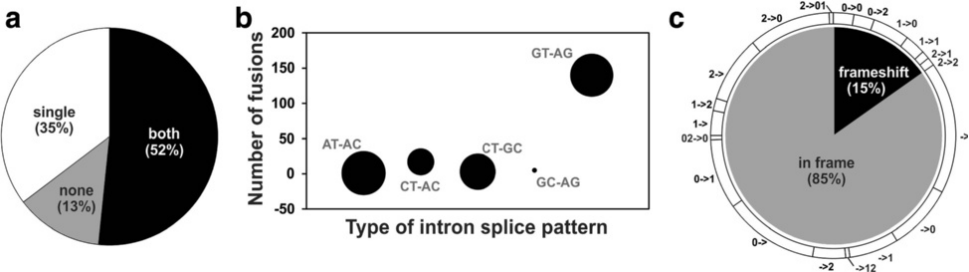
**a** Pie graph showing the proportion of fusion points that occur at the exon boundaries of one, both or neither genes that comprise the fusion transcript. **b** Bubble plot of the number of fusion transcripts that use the AT-AC, CT-AC, CT-GC, GC-AG, and GT-AG donor-acceptor splice sites. Bubble size represents the average gene expression (larger = greater expression) for fusion transcripts within that donor-acceptor class. **c** Pie chart of the percentage of fusion transcripts that maintain the original reading frames of the genes that comprise the fusion transcripts (inner pie chart). The outer pie chart represents the nucleotide position (0, 1, 2 = 1^st^, 2^nd^, and 3^rd^ nucleotide, respectively) within the codon of the first (*number before arrow*) and second (*number after arrow*) genes at the fusion points of those respective genes

An analysis of fusion junctions revealed that most fusion transcripts (160/185, 87 %) detected in our FusionMap analysis are formed at the exon borders of either one or both parental genes (Fig. 2a), and that most (140/185, 76 %) use the canonical GT-AG intron donor-acceptor splice sites (Fig. 2b). Interestingly, this observation also applied to fusion transcripts that are the result of chromosomal rearrangements such as TMPRSS2-ERG. Thus, fusion transcripts that are located within genomic regions that undergo genomic rearrangements still use the same transcriptional machinery as non-fusion loci, unless chromosomal breakpoints occur within exons. Notably, 50 % (12/24) and 70 % (46/66) of fusion transcripts that use the classical GT-AG intron donor-acceptor sites correspond to fusion junctions that were located at neither or only one exon boundary of the parental genes, respectively (Additional file 1). This indicates that these fusion transcripts are not the result of chromosomal breakpoints that occur within exons, but rather use classical gene transcription mechanisms to generate alternative exon boundaries. Importantly, 140 (85 %) of the fusion transcripts maintain the original open reading frame of the parental genes (Fig. 2c), opening the possibility that fusion transcripts can be translated into distinct functional proteins with unique biological properties. Indeed, 12 fusion proteins have already been detected in various human tissues [5].

### Computational prediction indicates that fusion junctions and non-fusion splice sites have similar propensities to hybridize

**Fig. 3:**
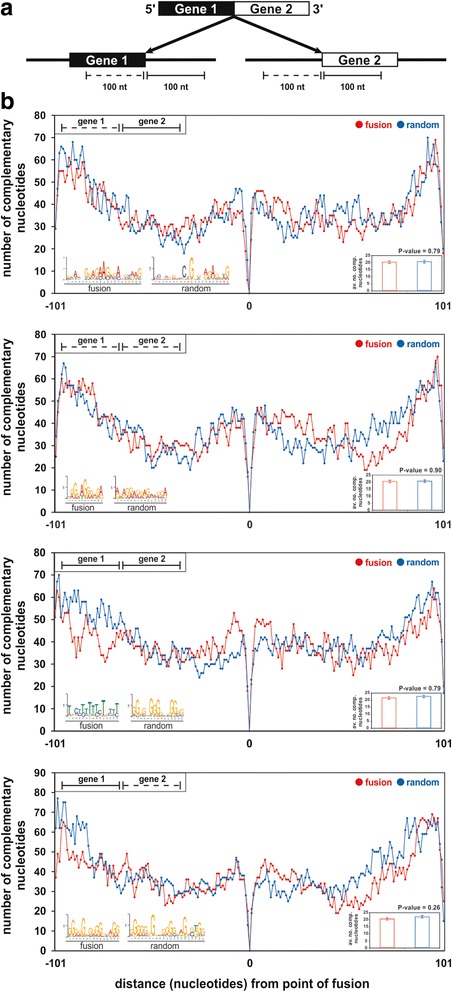
**a** Diagram showing 100 nt of genomic sequence upstream (*solid line* under gene) and downstream (*dotted line* under gene) of the point of fusion at the two genes comprising the fusion transcript that were used for hybridisation analysis. **b** The *line graph* represents the number of fusion transcripts that have complementary nucleotides (*y-axis*) at the respective distance (*x-axis*) from the point of fusion (*x-axis = 0*) between the up- and downstream sequences from gene 1 and gene 2. The histogram represents the average number of complementary nucleotides between the up- and down-stream sequences from gene 1 and gene 2. The MEME result (coloured ACGT nucleotides) represents motifs of complementary sequences between the up- and down-stream sequences from gene 1 and gene 2. Up- and down-stream sequences from random non fusion intron splice sites were used for comparison

It has been proposed that fusion transcripts might be the result of ‘chromosomal looping’ that brings distal loci together [25]. Thus, a computational analysis of the sequences flanking fusion junctions of the parental gene loci was performed to determine the capacity of these two loci to hybridize (Fig. 3a and Additional file 2), thereby bringing distal regions together to mediate one continuous transcriptional event that produces a fusion transcript. Our hypothesis stems from the RNA splicing process which similarly involves a series of steps comprising multiple nucleotide hybridizations between snRNA/ribonucleoprotein complexes with the target pre-mRNA [26]. We found no obvious difference in both the regions of hybridization, and the total number of hybridized sequences between parental gene loci of fusion transcripts (Fig. 3b, red lines) compared to canonical exon-exon boundaries of genes from the NCBI RefSeq database (Fig. 3b, blue lines). MEME analysis was also undertaken to assess for motifs that might promote genomic hybridization between parental gene loci. The predicted hybridized nucleotides of two gene 1/gene 2 combinations have different motifs between fusion and non-fusion loci (Fig. 3b, top panel, and third panel from top), while predicted hybridized nucleotides between gene 1 upstream and gene 2 upstream had G- and A-rich motifs (Fig. 3b, second panel from top). Notably, predicted hybridized nucleotides between gene 1 downstream and gene 2 upstream had similar G-rich motifs in both fusion and non-fusion loci (Fig. 3b, bottom panel).

### Fusion loci are depleted of repetitive sequences

**Fig. 4:**
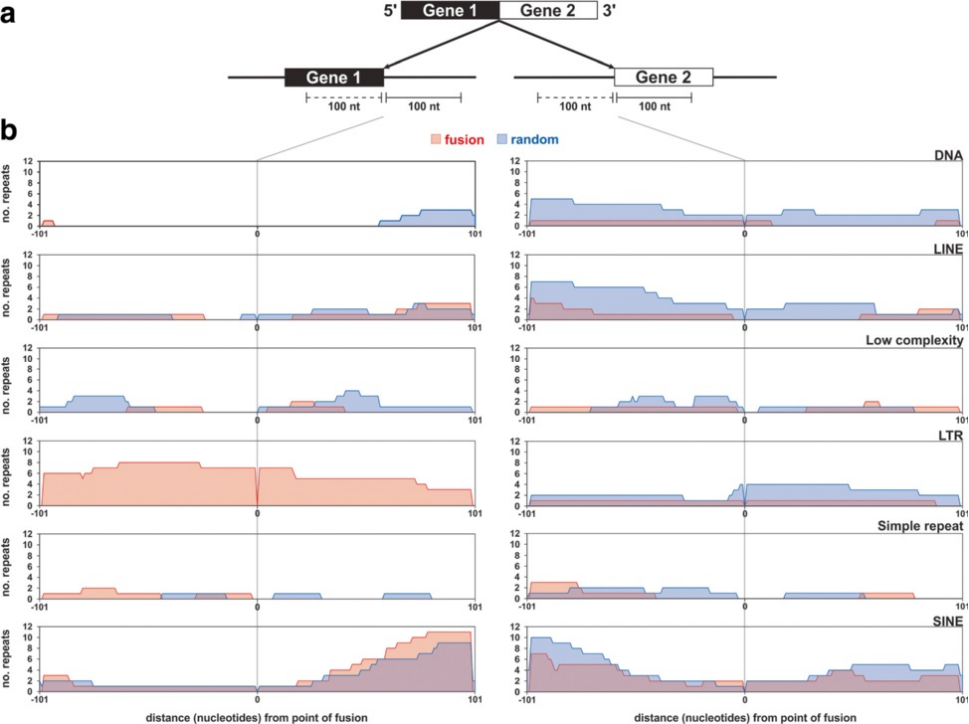
**a** Diagram showing 100 nt of genomic sequence upstream (*solid line* under gene) and downstream (*dotted line* under gene) of the point of fusion at the two genes comprising the fusion transcript that were used to identify repetitive sequences. **b** Repeats from six families (DNA, LINE, Low complexity, LTR, Simple repeat, SINE) were detected at fusion (*red regions*) and non-fusion (random, *blue regions*) regions at both gene loci

We also assessed whether there was a selection of repetitive sequences at fusion loci as it has been found that *Alu* repeats mediate aberrant gene transcription through *exonization* [27]. Sequence analyses of genomic sequences flanking the fusion junctions (red blocks) at parental gene loci (Fig. 4a) indicate that they have a low abundance of repetitive sequences. For example, only six repeat families were found within these regions (DNA, LINE, low complexity, LTR, simple repeat, SINE) (Fig. 4b). The highest prevalence of repeats corresponds to SINEs that were predominantly located further away from the point of fusion, but these account for only 6.5 % (11/168) of all fusions (Fig. 4b and Additional file 3). Apart from LTRs at the gene 1 parental loci, the number of repetitive sequences at non-fusion loci (blue blocks) was generally similar to parental gene loci of fusion transcripts (Fig. 4b). Given the likely use of similar transcriptional mechanisms between fusion and non-fusion loci from aforementioned observations, the lack of repetitive sequences at fusion loci is not surprising as exonic and splice regions are generally well conserved [28] to ensure functionality of important genes.

### Fusion transcripts are regulated by androgen and/or therapeutic anti-androgens

We assessed whether fusion transcript expression correlates with the parental gene expression in order to better understand the coordination of fusion expression with their parental genes. Thus, an RT-qPCR analysis was undertaken on six fusion transcripts that were in frame, and from frameshift classes with the most fusion transcripts. These include *CTBS-GNG5* (2→0), *DHRS1-RABGGTA* (0→), *SIDT2-TAGLN* (2→), *HARS2-ZMAT2* (→0), *NOS1AP-C1orf226* (0→1), and *C17orf106-CDK3* (→). Three of these fusion transcripts comprise of genes that are adjacent to each other in the genome (*HARS2-ZMAT2*, *DHRS1-RABGGTA*, *SIDT2-TAGLN*), with the other three derived from non-adjacent genes (*NOS1AP-C1orf226*, *C17orf106-CDK3*, *CTBS-GNG5*). RT-qPCR was also carried out on the *C1QTNF3-AMACR* (1→1) as a representative transcript with an interrupted reading frame. Our analysis indicates that all candidate fusion transcripts are either two-fold higher- (*DHRS1-RABGGTA*, *CTBS-GNG5*, *C17orf106-CDK3*, *SIDT2-TAGLN*) or lower- (*NOS1AP-c10rf226*, *HARS-ZMAT2*, *C1QTNF3-AMACR*) expressed after androgen or anti-androgen treatment in LNCaP cells (Table 1 and Additional file 4). In many cases, the androgen and anti-androgen regulation of at least one of the parental genes is similar to the fusion transcript (Table 1 and Additional file 4). *C1QTNF3-AMACR* was the notable exception (Table 1 and Additional file 4). The similarity in expression between fusion transcripts and their parental genes is not surprising considering that the splice site usage at fusion junctions is similar to those of non-fusion transcripts. Interestingly, all seven candidate fusion transcripts were regulated by androgen (DHT) and/or therapeutic anti-androgens (bicalutamide, enzalutamide), indicating that they might be important in disease progression and/or treatment resistance.

**Table 1:** Summary of fusion expression by RT-qPCR

		(anti)-androgen regulation^a^			Tumor expression^a^
Fusion transcript	Gene	DHT	BIC	ENZ	T/N
*NOS1AP-c1orf226*	Fusion	↓	↓	↓	-
	*NOS1AP*	↓	↓	↓	-
	*c1orf226*	↓	↓	↓	-
*HARS-ZMAT2*	Fusion	↓	-	-	-
	*HARS*	↓	↓	↑	-
	*ZMAT2*	↓	↓	-	↓
*DHRS1-RABGGTA*	Fusion	↑	↓	-	↓
	*DHRS1*	-	-	-	-
	*RABGGTA*	-	-	-	-
*CTBS-GNG5*	Fusion	↑	↑	↑	NE
	*CTBS*	↑	↑	↑	-
	*GNG5*	-	-	-	-
*C17orf106-CDK3*	Fusion	↑	↑	-	NE
	*C17orf106*	↑	↑	↑	NE
	*CDK3*	↑	↑	-	-
*SIDT2-TAGLN*	Fusion	↑	↑	↑	NE
	*SIDT2*	↑	↑	↑	↓
	*TAGLN*	↑	↑	↑	↓
*C1QTNF3-AMACR*	Fusion	↓	↓	↓	-
	*C1QTNF3*	↓	-	-	-
	*AMACR*	↑	-	-	↑

^a^At least two-fold higher- (↑), lower- (↓), or no change (-) in expression after (anti)-androgen treatment to mock treatments

^b^At least four of seven tumours with at least 2-fold over- (↑), under- (↓), or no change (-) in expression in tumors relative to non-tumor cells

*DHT* dihydrotestosterone (androgen), *BIC* bicalutamide (anti-androgen), *ENZ* enzalutamide (anti-androgen), *NE* not expressed

### Some fusion transcripts are differentially expressed in prostate tumors

Of the seven candidate fusion transcripts, only four (*NOS1AP-c10rf226*, *HARS-ZMAT2*, *DHRS1-RABGGTA*, *C1QTNF3-AMACR*) could be readily detected in clinical prostate tumors and/or adjacent non-cancer cells (Table 1 and Additional file 5). Of these, *C1QTNF3-AMACR* has an expression profile that is distinct from both parental genes (Table 1 and Additional file 5). Interestingly, the *DHRS1-RABGGTA* fusion transcript is less expressed in tumors compared to adjacent non-cancer cells (Table 1 and Additional file 5). Furthermore, five of the fusion transcripts detected in this study (*NOS1AP-c10rf226*, *HARS-ZMAT2*, *DHRS1-RABGGTA*, *CTBS-GNG5*, and *SIDT2-TAGLN*) were found in both our clinical RNAseq dataset comprised of Caucasian men, and in Ren and colleagues dataset which comprised of Han Chinese men [29]. Thus, these fusion transcripts are great candidates for further studies as they are readily expressed in different ethnicities.

### Fusion loci undergo extensive alternative transcription

**Fig. 5:**
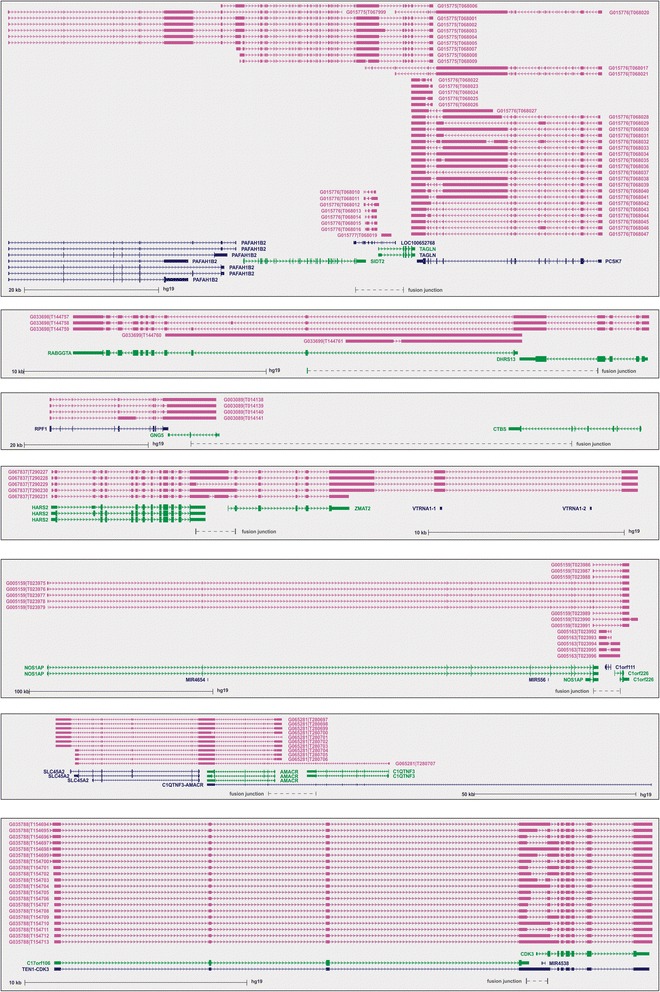
Diagram of other fusion transcripts expressed at the seven candidate fusion loci. Red UCSC Bed tracks indicate fusion transcripts discovered by Iyer et al. [19]. Parental genes that fusion transcripts were derived in our study are represented as green tracks, and other genes located at that locus are represented as blue tracks. The fusion junctions discovered in this study is also shown

Finally, we assessed for variant fusion transcripts given that most loci undergo variant transcription [19]. A recent large-scale RNAseq study comprising 7256 libraries from multiple cancers [19] was interrogated, revealing that 61 transcripts harbored the same exon junctions as the transcripts detected in our prostate (cancer) data set (Additional file 6). These 61 transcripts accounted for only 17 of the 185 fusion transcripts detected in this study (Additional file 6). This indicates that multiple variant fusion transcripts use the same exon junctions. In agreement, in addition to the 17 transcription-mediated fusion transcripts of our prostate-derived dataset, the Iyer et al. [19] dataset revealed that parental loci were extensively spliced, with 124/168 presenting alternative fusion transcripts (Additional file 1). We thus propose that these loci are highly amendable to fusion and alternative transcription. An example of extensive fusion transcription from the Iyer et al. dataset for the seven candidate fusion loci from this study is shown in Fig. 5.

## Conclusions

Using a conservative filtering process, we have identified 185 high confidence fusion transcripts that are readily expressed in prostate (cancer) cells in order to accurately analyze the sequences around fusion junctions in order to better understand fusion transcription and/or splicing. We reveal that fusion splices sites generally use the same nucleotide sequences as non-fusion transcripts, thereby indicating that fusion transcription likely co-opts the same transcriptional processes. However, this hypothesis may only apply to “read-through” chimeras which comprise the majority (76 %) of fusions that were detected in this study. Regardless of the mechanism, the recombination of exons from biologically distinct genes provides an interesting source of genetic variation that warrants further study which will further our understanding of the transcriptional nuances of more evolutionary complex species such as humans.

## Methods

### Ethical approval and consent to publish

All men have given written informed consent to the Australian Prostate Cancer BioResource to use their tissue and associated medical records for this study, as well as for publication of de-identified patient data. This study is also approved by the Queensland University of Technology Ethics committee (1000001165).

### Clinical prostate cancers

FFPE blocks from prostate tumors and their adjacent non-cancer cells were obtained from the Australian Prostate Cancer Bio-Resource tumor bank. Cells were extracted from formalin-fixed and paraffin-embedded sections of micro-dissected benign and malignant prostate tissues. Tissue blocks containing the tumor cells were serially sectioned (20 μm sections), transferred to glass slides, stained with methyl green, and tumor areas were marked and Gleason grades scored by a pathologist (Additional file 7). Marked areas were manually macro-dissected under a microscope using a sterile injection needle (size 0.65 × 25 mm). RNA was extracted using the miRNeasy FFPE kit (QIAGEN, Chadstone, Australia).

### RNA sequencing

RNA from eight clinical prostate tumors and adjacent non-cancer cells was sequenced by the Australian Genome Research Facility (http://www.agrf.org.au/). Briefly, ribosome-depleted RNA was paired-end sequenced on the Illumina HiSeq 2500 platform using 100 nt read lengths, and using the Illumina TruSeq strand-specific protocol (Life Technologies, Mulgrave, Australia). On average, 23.3 million reads were sequenced from each sample (Additional file 7).

### Detection of fusion transcripts

Fusion transcripts were identified using the FusionMap program [30] on the following RNAseq datasets: LNCaP prostate cancer cells treated with androgen (DHT) and therapeutic anti-androgens (bicalutamide, enzalutamide) [31], 14 clinical prostate cancers and their adjacent non-cancer cells [29], and from our RNAseq dataset of eight clinical prostate tumors and their adjacent non-cancer cells. FusionMap analysis was performed with raw RNAseq data files (FASTQ format), the Human. B37 reference genome and annotations, and default FusionMap parameters except for the following: PairedEnd = True, RnaMode = True, MinimalFusionAlignmentLength = 30, FusionReportCutoff = 1, NonCanonicalSpliceJunctionPenalty = 4. All predicted fusions were manually screened against the genome using the 30 nt fusion junction sequence from the FusionMap result and the UCSC genome browser BLAT tool [32]. Fusions mapping to several locations in the genome were discarded. The ‘SplicePatternClass’, ‘FrameShiftClass’, and ‘OnExonBoundary’ output fields from FusionMap were used to characterize the sequences at fusion loci.

Custom Perl scripts matching exon-exon junction co-ordinates from assembled bed and GTF files from a recent large-scale RNAseq study [19] against fusion junctions (gene 1 and gene 2 junction co-ordinates) from this study was used to identify common fusions between this study and from the Yu et. al study. Variant fusion transcripts at the 185 fusion loci from this study were detected by manual inspection using Yu and colleagues UCSC MiTranscriptome browser (http://mitranscriptome.org/).

### *In silico* base pair hybridization analysis of sequences flanking fusion splice sites

The computational workflow for analyzing hybridization of gene 1 and gene 2 genomic sequences is detailed in Additional file 2A. Briefly, 100 nt of genomic sequence up- and down-stream of fusion splice sites at genes composing the fusion transcript were obtained using a custom Perl script and RefSeq sequences. The up- or down-stream sequences of gene 1 were concatenated to the up- or down-stream sequences of gene 2 using a spacer of 20 ‘N’ nucleotides. The four combinations of sequences subjected to complementary sequence analysis are as follows: upstream gene 1--N_20_--upstream gene 2, upstream gene 1--N_20_--downstream gene 2, downstream gene 1-N_20_--upstream gene 2, downstream gene 1--N_20_-- downstream gene 2 (Additional file 2B). The DNA strand used for the sequences corresponds to the ‘strand’ output field from FusionMap. These four sequences were then analyzed for sequence hybridization using RNAfold which can use single-stranded DNA inputs [33]. A custom Perl script was then used to filter for nucleotides that hybridized between gene 1 and gene 2 sequences, as opposed to nucleotides that hybridized within gene 1 or gene 2 sequences (Additional file 2C). These hybridized sequences between gene 1 and gene 2 were then concatenated, and sequences comprising at least eight nucleotides were subjected to MEME analysis [34] to identify motifs. As a control, 185 non-fusion sequences from random consecutive exon splice sites of random genes (RefSeq genes) were used.

### *In silico* analysis for repetitive sequences at fusion loci

The 100 nucleotide sequences both up- and down-stream of fusion loci from above was also subjected to in *silico analysis* for the presence of repetitive DNA sequences which might mediate fusion transcription. However, for simplicity, only fusion transcripts from the same chromosome and which are not sense-antisense fusions (168 fusion transcripts) were chosen for analysis. A subset (168 of 185) of the 100 nucleotide sequences flanking random exons of random genes from above were also assessed to determine the baseline distribution of repetitive sequences at non-fusion loci. Repetitive sequences were defined by the RepeatMasker library (hg19.fa.out, Repeat Library 20120124, http://www.repeatmasker.org/). The prevalence of repetitive DNA near fusion and non-fusion splice sites where determined if the start and end coordinates of repetitive DNA overlapped with the start and end coordinates of the 100 nucleotide flanking sequences.

### Cell culture and RT-qPCR

The androgen receptor positive, LNCaP prostate cancer cell line was treated with androgen (10 nM DHT) (Sigma-Aldrich, Sydney, Australia), or therapeutic anti-androgens (10 μM bicalutamide, 10 μM enzalutamide) (Selleck Chemicals, Waterloo, Australia) for 48 h as described previously [31]. RNA was extracted from cells using Tri-reagent (Life Technologies), and reverse transcribed (RT) using Superscript III (Life Technologies) as described [35]. Quantitative PCR (qPCR) was carried out using SYBR Green master-mix (Life Technologies) using primers detailed in Additional file 8. Fusion expression was determined using the delta-delta CT method and using 18S as the house-keeping gene. Data is represented as the mean plus standard error from three independent RNA. A student’s t-test was used to test for significant differences in expression between mock and (anti)-androgen treated cells. RT-qPCR was also carried out as above on cDNA generated from seven tumor samples and adjacent non-cancer prostate cells.

## Additional files

Additional file 1:
**Summary of genomic features at fusion loci.** (XLSX 76 kb) Additional file 2:
**(A)** **Workflow of fusion analysis.** **(B)** Strategy for identifying nucleotide hybridisation between gene 1 and gene 2 at fusion splice sites. **(C)** Strategy to determine whether hybridization results from RNAfold are from within gene 1 and gene 2 sequences, or between them. Left “(“and right”)” brackets represent hybridised sequences. Innermost brackets are first matched (1, 2, 3, 4) to filter out hybridizations within sequences. Outermost brackets (5, 6, 7, 8) are then matched to identify hybridizations between sequences. N20 = linker spacer sequence. (JPG 1688 kb) Additional file 3:
**Repetitive sequences at fusion loci.** (XLSX 87 kb) Additional file 4:
**RT-qPCR analysis of (anti)-androgen regulation of** **seven candidate fusions** (***NOS1AP-C1orf226, HARS2-ZMAT2, DHRS1-RABGGTA, CTBS-GNG5***, ***C17orf106-CDK3, SIDT2-TAGLN***, ***C1QTNF3-AMACR***) **in LNCaP prostate cancer cells.** LNCaP cells were treated with either ethanol (Mock), 10 μM anti-androgens (bicalutamide (BIC), enzalutamide (ENZ)), or 10 nM androgen (DHT) for 24 h. Data is represented as the SEM from 2–3 independent RNA. Top panel = fusion transcripts, middle and bottom panels = parental genes that fusions were derived. (JPG 1528 kb) Additional file 5:
**RT-qPCR analysis of differential expression of the seven candidate fusions between tumours and** **adjacent** **non-cancer** **prostate cells in a cohort** (***n*** **= 7) of clinical prostate samples.** Histograms above 1, or below −1 represent a two-fold over- or under-expression in tumors compared to adjacent non-cancer cells, respectively. Top panel = fusion transcripts, middle and bottom panels = parental genes that fusions were derived. (JPG 1877 kb) Additional file 6:
**Fusion transcripts common to both Yu et al. dataset and from this study.** (XLSX 21 kb) Additional file 7:
**Patient data.** (XLSX 10 kb) Additional file 8:
**Primer sequences used in** **RT-qPCR.** (XLSX 11 kb)
