# Erratum to: Fusion transcript loci share many genomic features with non-fusion loci

**DOI:** 10.1186/s12864-016-2751-x

**Published:** 2016-06-03

**Authors:** John Lai, Jiyuan An, Inge Seim, Carina Walpole, Andrea Hoffman, Leire Moya, Srilakshmi Srinivasan, Joanna L. Perry-Keene, Australian Prostate Cancer Bioresource, Chenwei Wang, Melanie L. Lehman, Colleen C. Nelson, Judith A. Clements, Jyotsna Batra

**Affiliations:** Australian Prostate Cancer Research Centre – Queensland, Translational Research Institute, Brisbane, Australia; Cancer and Molecular Medicine Program, Institute of Health and Biomedical Innovation, Queensland University of Technology, Brisbane, Australia; Comparative and Endocrine Biology Laboratory, Institute of Health and Biomedical Innovation, Brisbane, Australia; Ghrelin Research Group, Institute of Health and Biomedical Innovation, Brisbane, Australia; Anatomical Pathology, Pathology Queensland, Brisbane, Australia; Current address: Genetic Technologies, 60-66 Hanover Street, Melbourne, Australia

## Erratum

After publication of the original article [1], a reader noted that one reference cited in the main text had not been mentioned in the References section. The reference (Qin et al., [2]) was cited as Ref. 33 within the text, but mistakenly did not appear in the References. As such the total number of References was also incorrect – there should have been 36 in total. References 33 – 35 should have been numbered 34 – 36 in the main text and in the References section.
