# Supplementary figures and images for: Fusion transcript loci share many genomic features with non-fusion loci
Source: BMC Genomics. 2015 Dec 1;16:1021. doi: 10.1186/s12864-015-2235-4 (PMC4667522; doi:10.1186/s12864-015-2235-4)

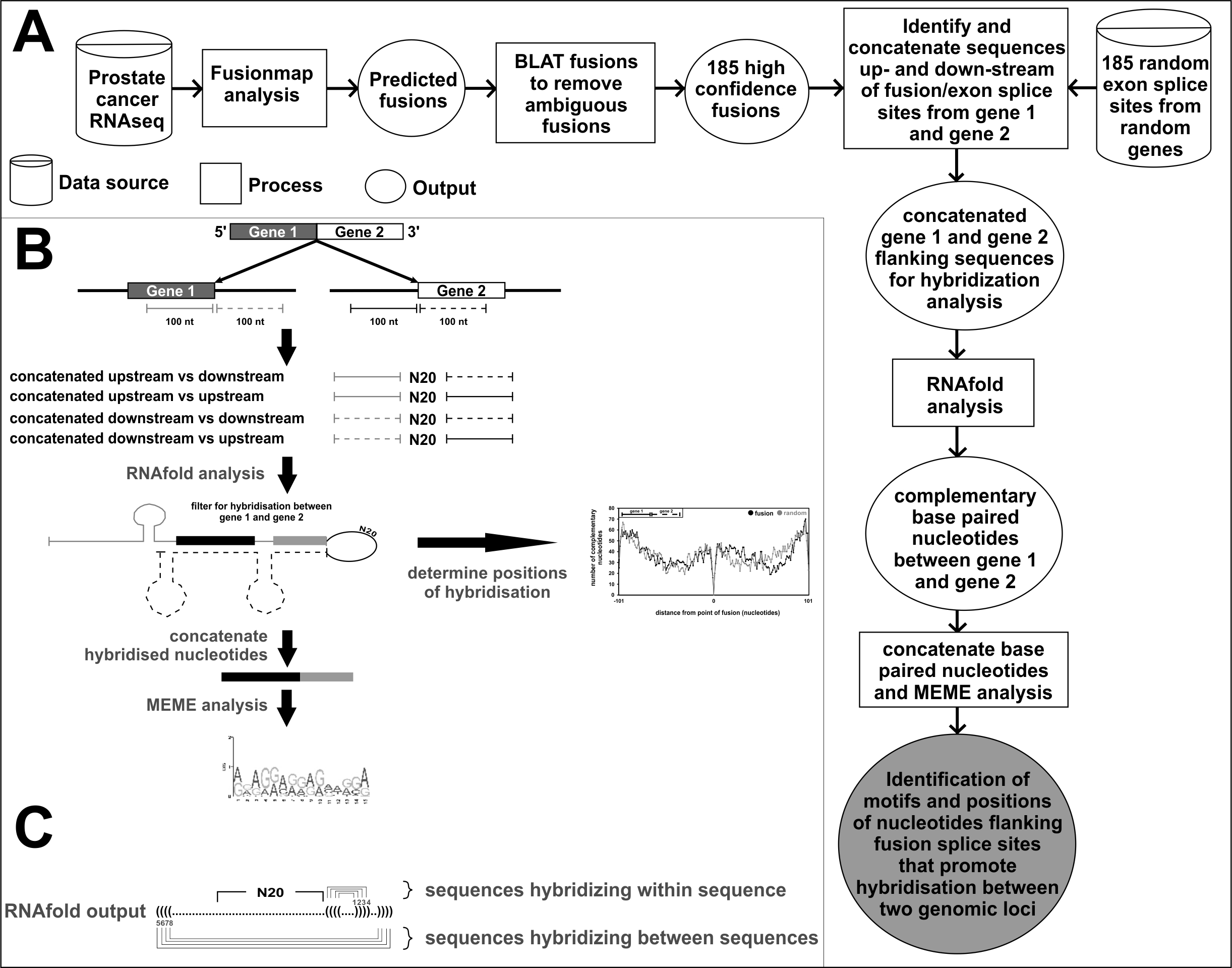

Supplement: Additional file 2: — (A) Workflow of fusion analysis. (B) Strategy for identifying nucleotide hybridisation between gene 1 and gene 2 at fusion splice sites. (C) Strategy to determine whether hybridization results from RNAfold are from within gene 1 and gene 2 sequences, or between them. Left “(“and right”)” brackets represent hybridised sequences. Innermost brackets are first matched (1, 2, 3, 4) to filter out hybridizations within sequences. Outermost brackets (5, 6, 7, 8) are then matched to identify hybridizations between sequences. N20 = linker spacer sequence. (JPG 1688 kb) [file 12864_2015_2235_MOESM2_ESM.jpg]

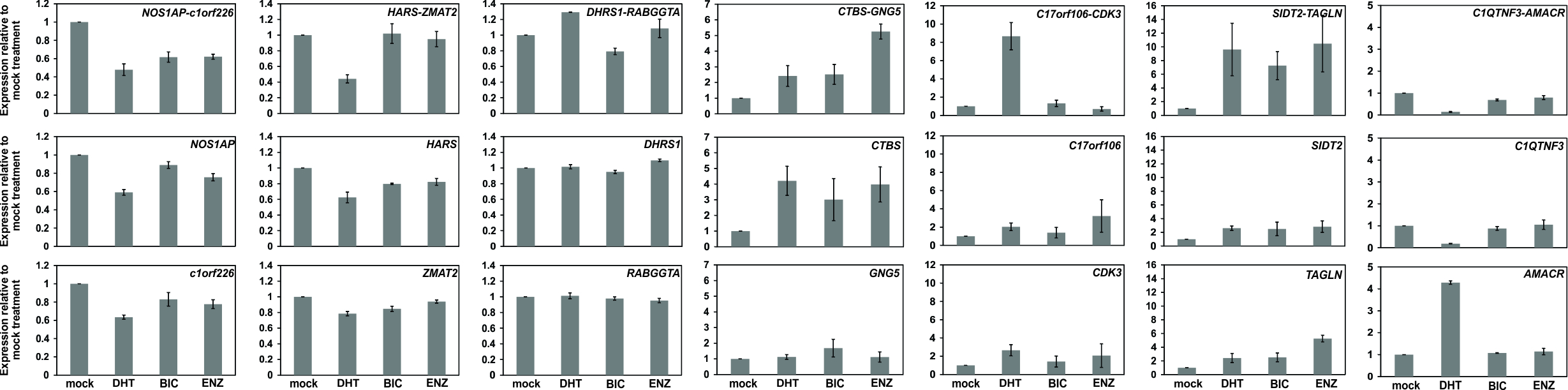

Supplement: Additional file 4: — RT-qPCR analysis of (anti)-androgen regulation of seven candidate fusions (NOS1AP-C1orf226, HARS2-ZMAT2, DHRS1-RABGGTA, CTBS-GNG5, C17orf106-CDK3, SIDT2-TAGLN, C1QTNF3-AMACR) in LNCaP prostate cancer cells. LNCaP cells were treated with either ethanol (Mock), 10 μM anti-androgens (bicalutamide (BIC), enzalutamide (ENZ)), or 10 nM androgen (DHT) for 24 h. Data is represented as the SEM from 2–3 independent RNA. Top panel = fusion transcripts, middle and bottom panels = parental genes that fusions were derived. (JPG 1528 kb) [file 12864_2015_2235_MOESM4_ESM.jpg]

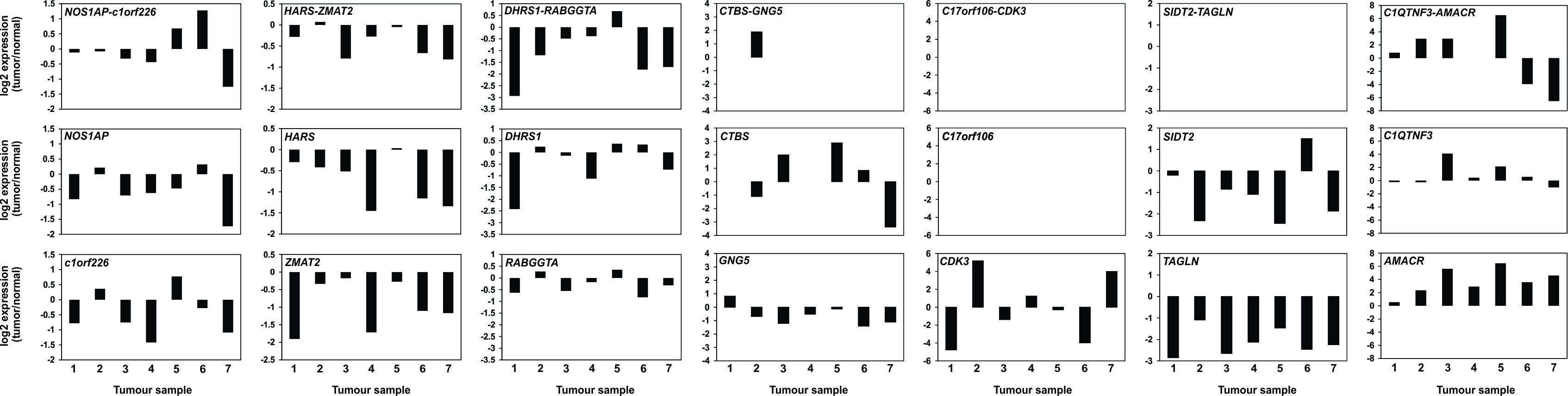

Supplement: Additional file 5: — RT-qPCR analysis of differential expression of the seven candidate fusions between tumours and adjacent non-cancer prostate cells in a cohort (n = 7) of clinical prostate samples. Histograms above 1, or below −1 represent a two-fold over- or under-expression in tumors compared to adjacent non-cancer cells, respectively. Top panel = fusion transcripts, middle and bottom panels = parental genes that fusions were derived. (JPG 1877 kb) [file 12864_2015_2235_MOESM5_ESM.jpg]
